# 625. Association of Eosinophilia with Parasites in Rhode Island Refugees, 2015-2020

**DOI:** 10.1093/ofid/ofad500.691

**Published:** 2023-11-27

**Authors:** Margarita Cruz-Sanchez, Benjamin Gallo Marin, Marcela Osorio, Maranatha Teferi, Ann Ding, Matthew Lorenz, Ian C Michelow

**Affiliations:** Warren Alpert Medical School of Brown University, Providence, Rhode Island; Warren Alpert Medical School of Brown University, Providence, Rhode Island; Warren Alpert Medical School at Brown University, Providence, Rhode Island; Warren Alpert Medical School of Brown University, Providence, Rhode Island; Brown University, Central Falls, Rhode Island; Brown University, Central Falls, Rhode Island; Connecticut Children's and UConn School of Medicine, Hartford, Connecticut

## Abstract

**Background:**

Newly arrived refugees in the United States have high rates of parasitic infections that may contribute to morbidity and mortality. There is little consensus on testing for eosinophils to screen for parasites. We hypothesized that eosinophilia was a useful biomarker for various parasites that could help guide the management of refugees in the future.

**Methods:**

We performed a retrospective chart review of all pediatric and adult refugees attending the only three refugee clinics in Rhode Island from 01/2015 to 12/2020. Individuals whose initial evaluation was delayed or took place in another state were excluded. Data were systematically collated in RedCap, and descriptive statistics were performed.

**Results:**

Of 812 eligible refugees, 147 (18.1%) had eosinophilia ( ⪰ 450/uL). The majority of refugees (505, 62.2%) were from Africa, 242 (29.8%) from Asia, 32 (3.9%) each from the Americas, Europe, and 1 (0.1%) from Australia. Eosinophilia was mild (450-1499/uL) in 113 (76.9%), moderate (1500-4999/uL) in 30 (20.4%), and severe ( ⪰ 5000/uL) in 4 refugees (2.7%)(**Table 1**). The prevalence of symptoms ranged from none (bloody stools) to 17.1% (abdominal pain). 48 (32.7%) refugees with eosinophilia tested positive for a parasite in their stool by O&P and/or PCR assay. The most prevalent organisms were *Giardia* (n=25) and *Blastocystis hominis* (n=22). An additional 22 (15%) tested positive for *Schistosoma* or *Strongyloides* (**Table 2**). Forty-six (31.3%) refugees with eosinophilia were treated with antiparasitic agents. Sixty-three (42.9%) had no follow-up eosinophil tests. Of the 84 patients who did have monitoring of their eosinophilia during the first year after arriving in the US, eosinophilia resolved in only 53 (64.6%). One patient (0.7%) had a severe complication: *Strongyloides* hyperinfection syndrome. Five patients (3.4%) received alternative diagnoses that explained their eosinophilia, including eczema, myelofibrosis, and a drug allergy.

**Figure:**
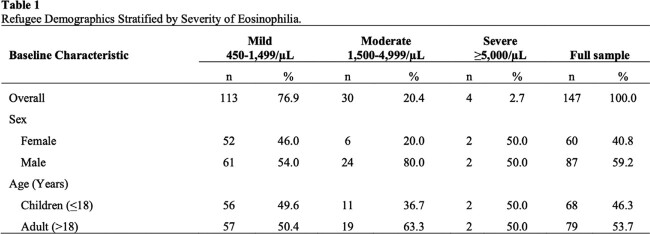

**Figure:**
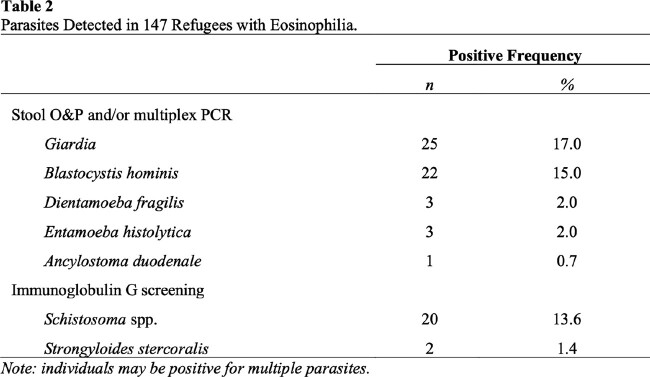

**Conclusion:**

Eosinophilia occurred in almost 1/5 of newly arrived refugees in RI. Diagnostic, therapeutic, and follow-up practices varied substantially. Approximately 1/3 of evaluable refugees had persistent eosinophilia at one year. A standardized screening approach for eosinophilia is needed to inform appropriate management.

**Disclosures:**

**All Authors**: No reported disclosures

